# Is Grassfed Meat and Dairy Better for Human and Environmental Health?

**DOI:** 10.3389/fnut.2019.00026

**Published:** 2019-03-19

**Authors:** Frederick D. Provenza, Scott L. Kronberg, Pablo Gregorini

**Affiliations:** ^1^Department of Wildland Resources, Utah State University, Logan, UT, United States; ^2^Northern Great Plains Research Laboratory, Agricultural Research Service (USDA), Mandan, ND, United States; ^3^Department of Agricultural Science, Lincoln University, Christchurch, New Zealand

**Keywords:** diet, nutrition, feedlots, grassfed beef, grazing, climate change, eat-lancet

## Abstract

The health of livestock, humans, and environments is tied to plant diversity—and associated phytochemical richness—across landscapes. Health is enhanced when livestock forage on phytochemically rich landscapes, is reduced when livestock forage on simple mixture or monoculture pastures or consume high-grain rations in feedlots, and is greatly reduced for people who eat highly processed diets. Circumstantial evidence supports the hypothesis that phytochemical richness of herbivore diets enhances biochemical richness of meat and dairy, which is linked with human and environmental health. Among many roles they play in health, phytochemicals in herbivore diets protect meat and dairy from protein oxidation and lipid peroxidation that cause low-grade systemic inflammation implicated in heart disease and cancer in humans. Yet, epidemiological and ecological studies critical of red meat consumption do not discriminate among meats from livestock fed high-grain rations as opposed to livestock foraging on landscapes of increasing phytochemical richness. The global shift away from phytochemically and biochemically rich wholesome foods to highly processed diets enabled 2.1 billion people to become overweight or obese and increased the incidence of type II diabetes, heart disease, and cancer. Unimpeded, these trends will add to a projected substantial increase in greenhouse gas emissions (GHGE) from producing food and clearing land by 2050. While agriculture contributes one quarter of GHGE, livestock can play a sizable role in climate mitigation. Of 80 ways to alleviate climate change, regenerative agriculture—managed grazing, silvopasture, tree intercropping, conservation agriculture, and farmland restoration—jointly rank number one as ways to sequester GHG. Mitigating the impacts of people in the Anthropocene can be enabled through diet to improve human and environmental health, but that will require profound changes in society. People will have to learn we are members of nature's communities. What we do to them, we do to ourselves. Only by nurturing them can we nurture ourselves.

## The Role of Livestock in Human and Environmental Health

**Figure 1:**
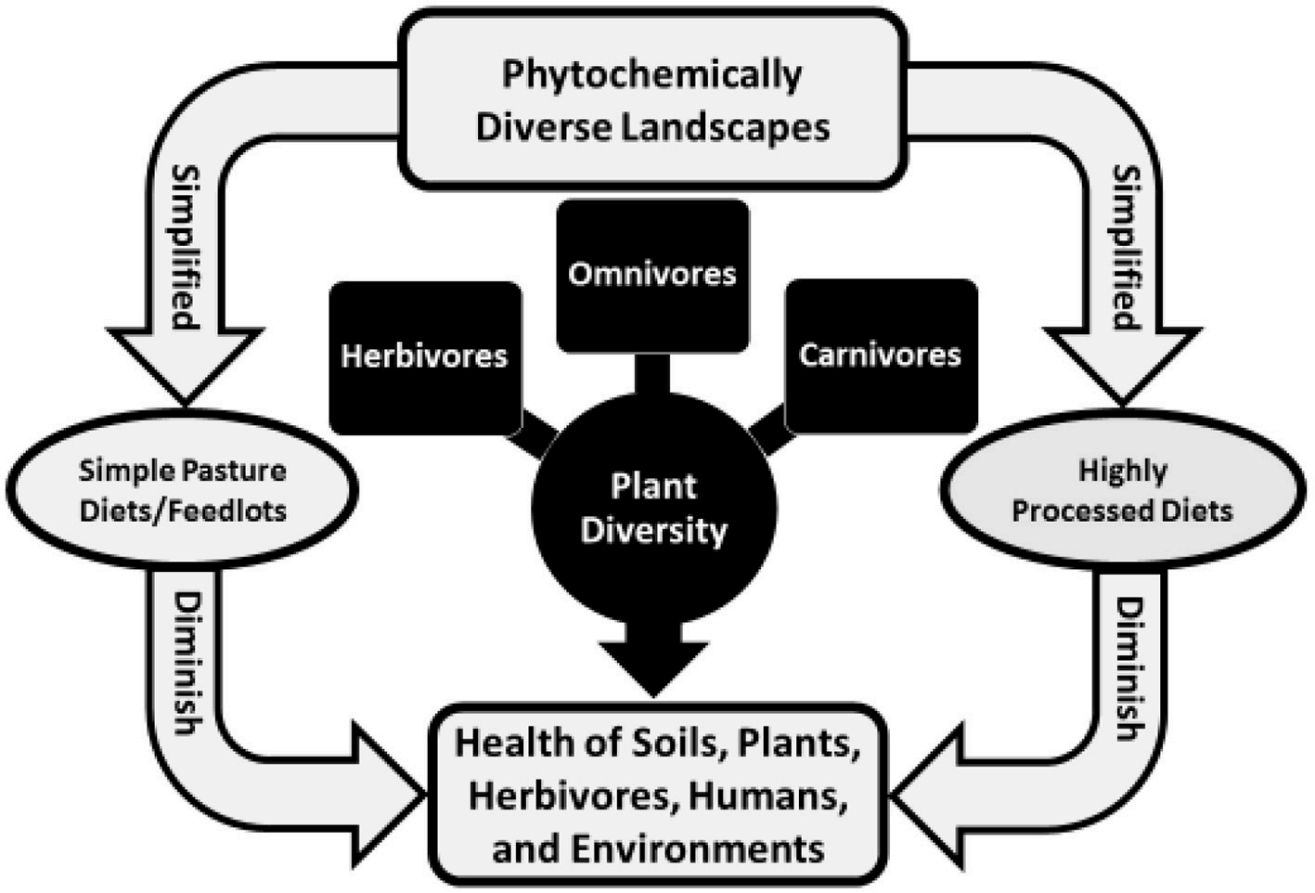
The health of life in soils, plants, herbivores, humans, and environments (land, water, and air) is tied to plant diversity—phytochemical richness—across landscapes.

Palates link the health of soil and plants with animals and biophysical environments. A palate attuned to a landscape enables herbivores and humans to meet needs for nutrients and to self-medicate (1). That evolves from three interrelated processes: biochemically mediated flavor-feedback associations where cells and organ systems, including the microbiome, alter liking for wholesome foods as a function of needs; accessibility to phytochemically and biochemically rich foods; and learning *in utero* and early in life to eat wholesome combinations of foods (2). That occurs when wild or domestic herbivores forage on phytochemically rich landscapes, is reduced when livestock forage on simple mixture or monoculture pastures or consume high-grain rations in feedlots, and is greatly reduced for people who eat highly processed foods obtained in contemporary food outlets (Figure 1).

Diets affect human and environmental health. The global shift to highly processed diets has enabled 2.1 billion people to become overweight or obese and increased incidence of type II diabetes, heart disease, and cancer (3–6). These trends have been amplified by primary health strategies focused on treating symptoms rather than preventing disease by promoting healthy diets and lifestyles (7). Unimpeded, these trends will add substantially to a projected 80% increase by 2050 in greenhouse gas emissions (GHGE) from food production (8).

Industrial agriculture uses for crops or pastures nearly half of the ice-free land on Earth, contaminates fresh and marine waters with nutrients and biocides, and contributes roughly one-quarter of the total GHGE from all economic activities (9). The input is larger in developing countries where agriculture and related land use activities can be more than half of total emissions (10). Growing human populations and demand for meat are increasing GHGE by agricultural practices dependent on fossil fuels and by converting tropical forests, savannas, and grasslands to crop and pasture lands, threatening many plant and animal species with extinction (11–13).

Some contend grain-based livestock finishing systems have less environmental impacts than forage-based grazing systems (14). While ruminant livestock begin their lives on pastures, nursing from their mothers and eating forages, only 4% of young animals continue to forage on pastures while the other 96% go to feedlots in the U.S. (15). Feedlots are characterized by controlled production practices that combine genetics, animal husbandry, and “nutritionally optimized” feeds to yield fat animals in less time than with grazing systems. That combination accelerates growth and enables more meat to be produced per unit area of land. Thus, Poore and Nemecek (16) claim for key metrics, such as land use and GHGE, feedlot systems generate fewer negative environmental impacts per unit of meat produced, especially for beef. Compared with feedlots, some pasture-finished beef production systems have markedly lower climate impacts, but pasture systems that require significant synthetic fertilization, inputs from supplemental feed, or deforestation to create pasture have substantially greater climate impacts than feedlot systems (17).

Others contend regenerative agriculture can reduce GHGE and sequester GHG, with added benefits that include enhanced biodiversity and ecological function. That occurs as damage to soil—from tillage, inorganic fertilizers, and biocides—is rectified with plant cover and animal manure that continually nurture soil in ways not possible with conventional production of crops grown to feed livestock in feedlots (18–23). Plant diversity and grazing are vital for maintaining healthy soil to sustainably grow grains in rotation with pastures on farmland (22, 24). Integrating livestock and perennial plants with food crops can restore soil and ecosystem health and increase yields (25). Moreover, farmlands can be managed to enhance biodiversity from microbes in soil to plants, insects, fish, birds, and mammals including livestock that contribute to production of wholesome foods, healthy soils, clean water, and sequestering GHG (26).

Managed grazing is a vital part of regenerative agriculture. At the highest level of sophistication, a skilled shepherd is an “ecological doctor” who has learned to use grazing to produce meat or milk and to create environmental health (27, 28). The herd in his or her hands is a living organism, biological and ecological “tools” for creating health of soil, plants, wild and domestic animals, and humans. Managed grazing can moderate climate change, an outcome that challenges the view of feedlots as the best way to reduce GHGE from livestock (29, 30). Collectively, managed grazing and other regenerative agricultural practices—silvopasture, tree intercropping, conservation agriculture, and farmland restoration—rank number one as ways to sequester GHG (31).

As opposed to pastures with few plant species and feedlots, health is enhanced when animals graze phytochemically rich mixtures of grasses, forbs, shrubs, and trees (32–37). Diverse plant communities are nutrition centers and pharmacies that enable health prophylactically and therapeutically (1). They are thus etiologic in the health of herbivores, omnivores, and carnivores above and below ground. Animals foraging on phytochemically diverse pastures require less anthelmintics and antibiotics than animals foraging on monoculture pastures or in feedlots. Overuse of antibiotics in feedlots adds to antibiotic resistance, a global health challenge (38, 39).

Yet, during the past 70 years, people have confined livestock in feedlots under conditions that violate the five freedoms of animal welfare (40, 41). They are moved from familiar social and biophysical environments (home) to unfamiliar environments (feedlots), which violates their freedom from fear and distress. Animals in feedlots are fed total-mixed rations high in grain with little chance to self-select their own diets, which violates their freedom to maintain individual health and vigor and produces changes in blood cortisol and behavioral parameters indicative of stress (42, 43). Individuals vary markedly in their preferences for different foods due to past experiences and individuality in morphology and physiology, which differentially affects their abilities to tolerate excesses and deficits of nutrients in their diets (44, 45). Animals acquire aversions to foods eaten too often or in excessive amounts (46, 47), and large numbers of animals confined and fed only total-mixed rations high in grain experience stress and malaise (nausea) (48), which violates their freedom from discomfort. To deal with cumulative effects on morbidity and mortality (49), animals are given antibiotics to counter illness from phytochemically impoverished diets and crowded conditions, which together violate their freedom from pain, injury, and disease.

Collectively, these practices, which have been scaled so people can afford to eat large amounts of grain-fed meat and dairy products, can be harmful for herbivores, humans, and environments (50–55). People in the U.S. eat meat and dairy at nearly three times the global average (56). Reducing intake of meat from feedlots, while increasing intake of meat from livestock finished on phytochemically rich landscapes, could reduce what some consider excessive intake of meat and increase intake of biochemically rich meat arguably of better quality, a key point not considered in the Eat-Lancet report (57).

While most livestock are fattened in feedlots in the U.S., and increasingly in other countries, those patterns are changing. In the U.S., for example, retail sales of pasture-finished beef have risen from $17 million in 2012 to $272 million in 2016 (15). That is 4% of beef sold and a market for pasture-finished beef that has grown at 100% annually for 4 years. People are also buying more dairy products produced from pasture (58). Interest in forage-fed meat and dairy is due to benefits for animal-welfare, consumer and environmental health, as well as authentication, terroir and geographical origin status.

Despite their alleged benefits, research has not elucidated linkages among plant diversity in herbivore diets and human health for either feedlot or pasture-based livestock production. Nor is plant diversity reflected in the generic label “grassfed,” which is why the flavors and biochemical characteristics of “grassfed” beef differ (59–61). In the absence of studies, we review circumstantial evidence that grazing systems have unrecognized benefits for health by addressing four questions: (1) Are specific compounds (e.g., omega-3 fatty acids) etiologic in human health? (2) Does the phytochemical richness of herbivore diets influence the biochemical richness of meat and dairy, and if so, does that affect the flavor and satiating characteristics of meat and dairy? (3) Does biochemical richness of meat and dairy affect human health? (4) How do diets of herbivores and humans influence environmental health?

## Biochemical Complexity and Human Health

Diet influences fatty acid profiles of animal tissues, and people often promote the health benefits of grassfed meat and dairy products based on improved ratios of omega-6 to omega-3 fatty acids (62, 63). Compared with diets high in cereal grains fed in intensive feeding systems, herbivore diets that are high in plants yield animal products that have higher levels of omega-3 fatty acids. Some scientists, medical doctors, nutritionists, and fitness advocates believe a healthy diet should have no more than 1–4 times more omega-6 than omega-3 fatty acids, but people who eat a diet high in processed foods consume a far higher ratio of omega-6 to omega-3 fatty acids (64). This imbalance is hypothesized to explain the increased incidence of heart disease, cancer, rheumatoid arthritis, autoimmune, and neurodegenerative diseases thought to stem from inflammation (65).

Increasingly in many nations, intake of the omega-6 linoleic acid comes from vegetable oils processed in ways that remove healthful components such as fiber, micronutrients, and many other phytochemicals present in unprocessed vegetables and seeds (66). Concentrated sources of linoleic acid are widely used as oils for cooking and added to processed and packaged foods. If these sources of linoleic acid are considered as supplements, people who eat diets high in processed foods are taking an equivalent of 11, 1-g capsules of linoleic acid daily over and above intake from wholesome foods. Yet, people who eat a processed diet, and ostensibly might benefit from less omega-6s, are unlikely to consume enough grassfed meat or dairy to offset their intake of omega-6s in other dietary items (67, 68).

Moreover, the benefits of consuming more omega-3 fatty acids and less omega-6 fatty acids are questionable. Historically, omega-6s were considered pro-inflammatory, but that was not the case in a review of randomized controlled clinical trials of the effects of the omega-6 linoleic acid on inflammation (69). Indeed, some studies attribute lower inflammatory markers to omega-6s (70). In an analysis of 20 prospective cohort studies from 10 countries, linoleic acid was associated with benefits for preventing type 2 diabetes and the omega-6 arachidonic acid was not harmful (71).

Interest in omega-3 fatty acids began with reports that Greenland Inuits, who ate a diet of oily fish and seal high in omega-3s, had low rates of cardiovascular disease (72, 73). Some researchers have questioned these findings because Bang et al. studied the diets of Inuits and only speculated that eating marine fats reduced cardiovascular disease (74). Other researchers emphasize Inuits had a prevalence of cardiovascular disease similar to non-Inuits; they had high mortality from cerebrovascular strokes; their general death rate was double that of non-Inuit peoples; and their life expectancy was roughly 10 years less than the Danish people Bang et al. used for comparisons (75). Nonetheless, reports by Bang et al. kindled great interest. Over 5,000 scientific papers—cited as evidence for the cardio-protective effect of the “Inuit Diet”—have explored the effects on health of omega-3s (75). Nutrition guidelines encourage people to eat fatty fish at least twice a week and to take supplemental omega-3s. Sales of omega-3 supplements are now a billion dollar industry and a marketing label for grassfed meat and dairy.

Yet, little evidence exists for the benefits of supplemental omega-3 fatty acids (75, 76). Initial trials with fish oil in Italy (77) and Japan (78) were encouraging, but subsequent studies cast doubts on their alleged benefits (79). Except for one trial (80), randomized, placebo controlled clinical trials have not shown protection against coronary events (81–86). Nor do supplemental omega-3s have any effect on the primary prevention of cardiovascular disease in people with diabetes (87). While they can improve heart function and reduce scarring after a heart attack (88), taking omega-3s preventatively does not lower risk of cardiovascular disease (89, 90), cancer (91), or all-cause mortality (92). In a meta-analysis of 10 trials, taking marine-derived omega-3s for an average of 4.4 years was not associated with reduced fatal or non-fatal coronary heart disease or major vascular events, stroke, cancer, or all-cause mortality (93). Nor does α-linolenic acid (ALA), the plant-derived precursor to eicosapentaenoic acid (EPA) and docosahexaenoic acid (DHA), reliably reduce risk of cardiovascular disease (94). Taking EPA-DHA or ALA did not decrease cardiovascular events for patients with a myocardial infarction who were receiving lipid-modifying, antihypertensive, and antithrombotic therapies (81). Neither EPA nor DHA retard macular degeneration (95) or slow memory loss (96–98). Some epidemiological studies suggest DHA is associated with less risk of Alzheimer's disease, but a complete account will require placing DHA in the context of the entire spectrum of omega-3 fatty acids (99).

These findings highlight often overlooked evidence that human health is enhanced as the biochemical richness of diets increases from compounds such as EPA or DHA, to mixtures of compounds such as omega-3s (100), to foods such as oily fish that contain hundreds of compounds in addition to omega-3 fatty acids (101), to mixtures of wholesome foods such as oily fish, meat and milk, vegetables and fruits that contain tens of thousands of bio-active compounds (102). Inconsistent findings among omega-3 trials are due in part to the simplicity of compounds—for example the simplicity of EPA, DHA, or ALA—relative to the synergies that occur among all of the omega-3 fatty acids (100). That is why supplements or foods with added omega-3s do not exhibit consistent benefits, yet increased intake of fish is associated with lower inflammatory responses in people with metabolic syndrome (103). That is also why current advice is to eat oily fish rather than take supplemental omega-3s (100).

Phytochemically rich diets for herbivores and biochemically rich diets for humans include not only primary compounds—such as energy, protein, minerals, and vitamins—but the tens of thousands of other so-called secondary compounds—including but not limited to phenolics, terpenoids, and alkaloids—that in moderate amounts can have health benefits (1, 2). While any primary or secondary compound can be toxic when ingested in too high amounts, they have health benefits when consumed in moderation and in combinations as part of phytochemically diverse diets for herbivores and biochemically diverse diets for humans (1, 45). Complementarities and synergies among primary and secondary compounds within and among meals promote health.

## Herbivore Diets Link Meat and Dairy With Human Palates and Health

By providing high-quality protein and essential micronutrients such as iron, zinc, and vitamin B_12_, meat is important in human nutrition. Nevertheless, some contend people now eat too much red meat and processed meat, which is associated in epidemiological (prospective cohort) studies with increased risk of cancer, cardiovascular and respiratory diseases, and type 2 diabetes (53, 104–106). Conversely, prospective cohort studies show reduced mortality from all causes in vegetarians (9%) and vegans (15%) compared with non-vegetarians (107), and reduced mortality of 12–20% in vegetarians compared with non-vegetarians (108).

These findings notwithstanding, a prospective cohort study of people in the United Kingdom found no reduction in mortality for vegetarians compared with non-vegetarians (109). In that study, both vegetarians and non-vegetarians had lower rates of mortality than the national average. Meat intake among non-vegetarians was a modest 79 g/d in men and 67 g/d in women, and intake of vegetables and fruit was only 20% higher for vegetarians than non-vegetarians. Eating fruits and vegetables with meat likely benefited the health of non-vegetarians.

Some contend eating too much red meat promotes oxidative stress and low-grade systemic inflammation—characterized by elevated plasma levels of pro-inflammatory markers such as C-reactive protein, serum amyloid A, tumor necrosis factor alpha, and interleukin 6—implicated in cancer, cardiovascular disease, metabolic syndrome, insulin resistance, and type 2 diabetes (110, 111). These diseases allegedly are due to ingesting excesses of compounds such as heme iron in red meat and nitrate/nitrite in processed meat (53, 112–115).

Inferring the health impacts of dietary patterns from epidemiological studies is problematical due to multiple confounding factors, many of which are not known or taken into account (116), including how the phytochemical diversity of herbivore diets affects the biochemical characteristics of meat and milk. Epidemiological studies that find inverse associations between eating red meat and health do not distinguish between meat from livestock fed high-grain diets in feedlots and livestock foraging on phytochemically rich mixtures of plants. Nor do they address how herbs, spices, vegetables, and fruits eaten in a meal with meat can enhance health.

Herbivore diets influence the flavor and biochemical richness of meat and dairy such that laboratory analyses can distinguish animals eating diets of increasing phytochemical richness, ranging from cereal grains to grain-pasture mixes to pastures (117). Among many other compounds, phenolics, carotenoids, and terpenoids in herbivore diets can enhance the flavor and biochemical characteristics of meat, fat, milk, and cheese (118). For example, tannins in herbivore diets improve the flavor of meat by reducing rumen bacteria that produce “off-flavors” from skatole, a mildly toxic organic compound produced from tryptophan in the mammalian digestive tract; tannins also affect rumen biohydrogenation of polyunsaturated fatty acids, which changes fatty acid profiles in meat (119). Adding garlic or essential oils from juniper, rosemary, or clove to the diets of lambs and calves improves the flavor of their meat and each of these plants contains a host of secondary metabolites that can benefit human health (120–122).

Phytochemical richness may be one reason why people have decidedly lower post-prandial inflammatory responses when they eat the meat of kangaroos foraging on diverse mixtures of native plants (a traditional hunter-gatherer meat meal) than when they the eat meat of wagyu cattle fed high-grain diets in feedlots (a modern meat meal) (123). Eating any food causes a transient post-prandial inflammatory response (124–126), and when people eat meat and fat, protein oxidation and lipid peroxidation cause inflammation (127). Yet, when herbivores eat phytochemically rich diets, compounds in their diets protect meat and dairy from the protein oxidation and lipid peroxidation that cause inflammation (128–130). In the study of kangaroos and wagyu cattle by Arya et al. (123), diet and animal were confounded and no studies have assessed how the phytochemical richness of forages herbivores eat affects the biochemical richness and flavor of their meat and fat and how that might affect inflammation.

Hunter-gatherers are noteworthy for their metabolic and cardiovascular health (131). They have less heart disease, cancer, diabetes, and osteoporosis than people who eat diets high in processed foods, and that is not because hunter-gatherers die before they develop these diseases (132). Although their diets are high in red meat, fat, and milk, the Maasai in southeastern Africa have less heart disease and cancer than do people who eat a diet high in processed foods (133). Nor are the diets of hunter-gatherers necessarily low in carbohydrates, as is often argued: the Hadza diet includes 16–20% honey, which is roughly 15% of their energy intake (131). Their low incidence of cardiovascular disease and obesity can be attributed in part by their higher levels of physical activity compared with people who eat diets high in processed foods (134, 135). The Maasai also add up to 28 herbs to meat-based soups and 12 herbs to milk (133). Diets of hunter-gatherers are also less energy dense and richer in fiber, micronutrients, and phytochemicals than processed diets. Findings from clinical trials and prospective cohort studies show relatively high intakes of dietary fiber and whole grains are complementary, and the prominent dose-response relationships with non-communicable diseases suggest the responses are causal (136).

Historically, Native Americans used wild berries—including but not limited to serviceberry (*Amelanchier alnifolia*), highbush cranberry (*Viburnum trilobum*), chokecherry (*Prunus virginiana*), and silver buffaloberry (*Shepherdia argentea*)—for food and medicine. Dried meat and fat were combined with berries to make pemmican, thus enabling use of dried berries during fall and winter. Berries contain rich arrays of phytochemicals that protect against metabolic syndrome, diabetes, diabetic microvascular complications, hyperglycemia, and pro-inflammatory gene expression (137). Compounds in berries improve metabolic syndrome by modulating lipid metabolism and energy expenditure. Berries contain polar compounds—proanthocyanidins, anthocyanins, and phenolic acids—that are hypoglycemic agents whose activities strongly inhibit IL-1β and COX-2 gene expression. Berries also contain non-polar compounds such as carotenoids that inhibit aldose reductase, an enzyme involved in diabetic microvascular complications. Eating fruits (and vegetables) reduces risks of type 2 diabetes, cardiovascular disease, cancer, and all-cause mortality (138, 139).

Eating antioxidant-rich fruits and vegetables with a high-fat meal improves vascular function and thwarts the negative effects of fat on endothelial function (140–142). Most plasma-borne markers of inflammation are not reliably raised after a high-fat meal, but they are reduced in many studies when meals include vegetables (143). While beneficial effects are related to antioxidant and anti-inflammatory properties, polyphenolic compounds also modulate cellular lipid metabolism and thus mitigate atherosclerotic plaque formation (144). People who eat polyphenol-rich foods, vitamin E, and calcium have less risk of colon cancer, evidently because these compounds protect against excess heme iron in red meat (145). Phytochemicals can reverse epimutations and counter all of the hallmarks of cancer (146, 147). Collectively, these studies suggest eating vegetables and fruits, along with meat, enhances health through biochemical interactions that occur within the body during a meal—with one caveat. People who eat large amounts of vegetables high in nitrates—such as beets, celery, lettuce, radishes, and spinach—along with processed meats high in nitrates may have greater risks of disease (53), though some contend the body of evidence suggests foods enriched in nitrate and nitrite provide health benefits with little risk (148).

Cooking hamburger can generate reactive oxygen species such as malondialdehyde (MDA), a marker for oxidative stress and inflammation (149). However, adding polyphenol-rich antioxidant spices to hamburger enhances flavor while reducing meat, plasma, and urine MDA levels (150). Herbs such as rosemary and oregano enhance flavor and inhibit lipid peroxidation (151). Postprandial plasma levels of MDA rise by 3-fold after a meal of red meat cutlets, but drinking polyphenol-rich red wine along with cutlets reduces levels of MDA by 75% (152). That is one reason why red wine and red meat complement one another. Polyphenols also counteract endothelial dysfunction in people fed a high-fat diet (153). Polyphenols added to a red-meat diet fed to rats prevents lipid peroxidation in the gut and absorption of MDA into the plasma (154).

While people must eat large amounts of food to meet needs for energy and protein, phytochemically rich herbs and spices added in trifling amounts to foods enhance palatability, satiation (when a meal ends), and satiety (length of time between meals) because herbs and spices are good for health (155, 156). People eat less when food provides more sensory pleasure than they do of a blander version of the food (157). For example, people prefer the flavor and satiate more rapidly when soup is spiced with chili compared to the base soup (158, 159). These flavor-feedback relationships occur as cells and organ systems, including the microbiome, respond to primary and secondary compounds in foods (1). Nevertheless, no research has assessed how palatability, satiation, and satiety are affected by the biochemical richness of meat or dairy.

Herbivore diets influence the flavors of milk and cheese (58). For example, cattle fed diets high in lipids produce sweet, raspberry-flavored γ-dodecalactone from oleic acid and sweet, raspberry-flavored γ-dodec-cis-6-enolactone from linoleic acid; cattle fed diets low in lipids produce milk fat high in cheesy-flavored fatty acids and precursors of the blue-cheese-flavored methyl ketones and coconut-peachy-flavored δ-lactones (160). Among many other compounds in forages, carotenoids impart a yellow color and they positively influence the flavor of milk and cheese. Terpenes also positively influence flavor of milk and dairy products derived from native pastures with diverse species of grasses, forbs, and shrubs that produce many more terpenes than do monocultures of grasses. Plant diversity also affects phenolics in cheeses such as L'Etivaz and Gruyere (161, 162).

When dairy cows graze botanically diverse swards, rather than a total-mixed ration of cultivated forages and grains, both the flavor and biochemical richness of their milk and cheese are greatly enhanced, and local peoples prefer the flavors of milk and cheese from dairy cows grazing on the botanically diverse swards (163, 164). Consumers in countries such as Italy and France select cheeses based on season of production and the related mix of plants in particular landscapes—for example, cheese made from high elevation summer pastures in the Alps—their palates linked locally with soil, plant diversity, and herbivore diets (165). Compared with trained evaluators, untrained evaluators, who typify naïve consumers, are less able to distinguish and savor differences in milk and cheese, which illustrates how past experiences influence palatability. More research is required to elucidate how herbivore diets affect biochemical richness and palatability of milk and cheese (58).

As with milk and cheese, people prefer meat they are accustomed to eating (166). When Spanish milk/concentrate-fed lambs and British grassfed lambs were assessed by Spanish and British taste panels, both panels found British lamb had higher flavor intensity, but the Spanish panel preferred milk/concentrate-fed lambs, while the British panel preferred grassfed lambs (167). Families in Mediterranean and Northern European countries—Greece, Italy, Spain, France, UK, and Iceland—also differ in their preferences for meat depending on whether they are accustomed to eating lambs fattened on grain or on pastures (168). Most Americans are conceived and raised eating grain-fed beef, so taste panels of consumers, as well as experts trained to evaluate sensory features of meat, typically find grain-finished beef more palatable than grass-finished beef (169–171). Inconsistent ratings for grass-finished beef in studies reflect differing past experiences of consumers and differences in how animals are finished. Collectively, these studies show why the generic label “grassfed” tells a consumer little about how the phytochemical richness of the diet contributes to flavor or health (59–61, 172, 173).

## Biochemically Rich Diets and Environmental Health

As humans transitioned from hunter-gatherers to farmers, ranchers, and urbanites, our diets shifted to include more highly processed foods, refined sugars and fats, and meat. How we produce food is adversely affecting food quality, both the phytochemical richness of herbs, spices, vegetables, and fruits and the biochemical richness of meats (1, 172), In turn, the foods we consume are adversely affecting health, as illustrated when researchers compared four diets (8): (1) Vegetarian—vegetables, fruits, grains, sugars, oils, eggs and dairy, and normally not over one serving a month of meat or seafood; (2) Pescetarian—vegetarian diet with seafood; (3) Mediterranean—vegetables, fruit, seafood, grains, sugars, oils, eggs, dairy and modest amounts of poultry, pork, lamb, and beef; and (4) Omnivorous—includes all food groups, for example the 2009 global-average diet and the income-dependent diet projected for 2050, which is essentially a diet that includes many processed foods high in refined carbohydrates, refined fats, oils, and meats.

Compared to the omnivore diet, the other three diets had a lower incidence of type II diabetes, (16–41%), cancer (7–13%), mortality from coronary heart disease (20–26%), and mortality from all causes combined (0–18%) (8). When a projected population increase to 9.7 billion people is combined with a projected increase of 32% in per person emissions from shifts to an omnivore diet, the net effect is an estimated 80% increase in global GHGE from food production by 2050. Alternatively, net GHGE from food production would not increase if the global diet was vegetarian, pescetarian, or Mediterranean. These diets could ostensibly reduce GHGE below those of the projected 2050 income-dependent diet, with reductions of 55, 45, and 30% for vegetarian, pescetarian, and Mediterranean diets, respectively. These findings are similar to other systematic reviews that assessed the impacts of diets on GHGE, land use, water use, and health (174).

Life cycle assessments suggest plant foods have less GHGE than do animal foods, and ruminant meats have greater GHGE per gram of protein than poultry, pork, eggs, dairy, non-trawling seafood, and traditional aquaculture (16, 175). Yet, those assessments generally do not address nuanced relationships among the health of soil, plants, herbivores, and humans (57, 176). When the environmental footprint—expressed both as land use for production and as GHGE—of plant and animal foods is calculated to consider essential amino acids in required amounts, animal foods are similar to most plant foods due to the higher quality of animal proteins (177). Grass-finished livestock can also promote nutrient cycling, soil carbon sequestration, and clean water and support food security (178–180).

Worldwide, agriculture involves 570 million farms and ranches—over 90% of them managed by a family and reliant on family labor—that produce 80% of the world's food (181). Agriculture employs over 1.3 billion people, nearly 40% of the global workforce (181). In nearly 50 countries, agriculture provides work for 50% of the population, up to 75% in poorer nations. Production of meat and dairy from cattle, sheep, and goats provides job security and food from animals that graze land unsuitable for farming and eat crop residues (179, 180). Livestock convert more than 432 billion kg of food/fiber byproducts inedible by humans into human-edible food, pet food, industrial products, and 4 billion kg of N fertilizer (182). In the U.S., 2.2 million farms and ranches cover 922 million acres; agriculture employs 1.6 million people and produces $31.8 billion in exports; and animal-derived foods provide considerable energy (24%), essential fatty acids (23–100%), protein (48%), and amino acids for people (34–67%) (182).

Due to low yields of beef from extensive grazing, people in Brazil are considering converting pastures to cropland for soybeans or to sugarcane for ethanol, but intensifying grazing can help meet projected 80% increases in demand for beef by 2050. Compared with crops, intensifying grazing management produces greater ecological benefits, including enhanced soil health and carbon sequestration (18, 55). Some nuances of these relationships are illustrated by comparing biological type of cattle—small (3) or large (5) frame sizes—and nutritional regime. Cook et al. (183) found that large-frame steers ate more forage, gained weight more rapidly, and were heavier at slaughter than small-framed animals when they were finished in feedlots, but when they were finished on forages, small-framed animals were in better body condition. Outcomes depended on nutritional regimen—finished in feedlots; fed native range (short-grass prairie in eastern Colorado) yearlong; fed native range complemented by crested wheatgrass in spring; fed native range accompanied by crested wheatgrass in spring and forage sorghum in late summer and winter. Grazing complementary forages increased beef production per hectare by 53% compared with grazing only native range. During a 97-day finishing period in feedlots, feed efficiency (kg feed/kg gain) and weight gain declined significantly during the last 31 days, a time when weight gain was mainly fat and little protein. Energy inputs lost in producing carcasses with excessive cut-away fat were important, as roughly 91% of the energy for feedlot finishing was for feed production. Compared with forages, feeding concentrates was expensive. The opportunity is to create grazing-based livestock-production systems based on phytochemically diverse forages for specific ecoregions at temporal and spatial scales that enhance livestock production and ecological services (184).

Of 80 ways to mitigate climate change, regenerative agriculture—managed grazing, silvopasture, tree intercropping, conservation agriculture, and farmland restoration—jointly rank number one as ways to sequester GHG. Silvopasture systems that combine growing trees with managed grazing rank ninth while managed grazing ranks nineteenth (31). The impacts of managed grazing are due to benefits that accrue through enhanced plant health and diversity over vast grazing lands (20, 185). Long-term storage of carbon in soil with silvopasture can be five times more than with managed grazing alone, not including carbon stored in trees (186–188). Silvopasture delivers efficient feed conversion, enhanced biodiversity, improved connectivity among habitats, and enhanced animal welfare (19). Grasses, forbs, and shrubs add resilience to silvopasture systems in the face of rising temperatures, drought, and fires, which are causing some forests, unable to cope with changing climates, to die and transform from carbon sinks to carbon sources (189). In addition to sequestering carbon, emissions of methane and nitrogen can be reduced when ruminant diets contain tannins and saponins common in forbs, shrubs, and trees (190–192). The notion that regenerative agricultural practices can markedly influence climate is consistent with evidence that carbon uptake from the atmosphere by native plants, which invaded abandoned farms following massive depopulation of the Americas following European arrival, contributed to global cooling during the Little Ice Age (193).

## Upshot

Circumstantial evidence supports the hypothesis that plant diversity—manifest as phytochemical richness of landscapes—affects the biochemical richness of meat and dairy as well as human and environmental health. Future studies should elucidate how plant diversity influences flavor and biochemical richness of meat and dairy; how phytochemically rich herbs, spices, vegetables, and fruits complement meals that contain meat; and how the aforementioned affect the health of people and the planet. Findings from these studies can achieve three ends. First, they can reveal relationships among liking for the flavor of meat and dairy; the ability of phytochemically and biochemically rich meals that contain meat and dairy products to satiate; and the value to cells and organ systems, including the microbiome, of phytochemically and biochemically rich foods for humans. Second, they will underscore why more money and effort ought to be spent creating human and environmental health by growing and eating wholesome foods and less effort spent treating symptoms of diet-related diseases. Finally, they will help people appreciate how the foods we eat reflect our relationships with land, water, and air, enabled by plant diversity across landscapes, thus revealing how palates link soil and plants with animals and environments.

While the Anthropocene is a curse for the havoc it is reeking globally on populations of plants and animals, including humans, it is a blessing because *Homo sapiens* may finally come to appreciate the crux of Aldo Leopold's land ethic (194). We are members of natural communities: what we do to them, we do to ourselves. Only by nurturing them can we nurture ourselves. Palates link cultures with landscapes and moderating the impacts of palates on human and environmental health will require changes in the kinds of foods we produce and consume, how we produce food, and how we reduce food waste, which is 40% of food produced annually and a major contributor to GHGE (31, 195–197). That will necessitate collaboration among food producers, food industry, nutritionists, ecologists, health professionals, educators, and policy makers with support of consumers. Forsaking diets high in processed foods will be challenging, but that can be facilitated if consumers appreciate the influence of diet on human and environmental health (16). These transformations can occur socially, economically, and ecologically by growing wholesome foods—plants and animals—as the basis for meals that nourish the health of people and the planet (1, 21, 22, 24).

## Author Contributions

All authors listed have made a substantial, direct and intellectual contribution to the work, and approved it for publication.

### Conflict of Interest Statement

The authors declare that the research was conducted in the absence of any commercial or financial relationships that could be construed as a potential conflict of interest.
